# Differential regulation of intramuscular fat and abdominal fat deposition in chickens

**DOI:** 10.1186/s12864-022-08538-0

**Published:** 2022-04-15

**Authors:** Na Luo, Jingting Shu, Xiaoya Yuan, Yuxi Jin, Huanxian Cui, Guiping Zhao, Jie Wen

**Affiliations:** 1 State Key Laboratory of Animal Nutrition, Institute of Animal Sciences, Chinese Academy of Agricultural Sciences, Beijing, 100193 China; 2 Key Laboratory for Poultry Genetics and Breeding of Jiangsu Province, Poultry Institute, Chinese Academy of Agricultural Sciences, Yangzhou, 225125 China

**Keywords:** Abdominal fat, Chicken, Intramuscular fat, Transcriptomics, WGCNA

## Abstract

**Background:**

Chicken intramuscular fat (IMF) content is closely related to meat quality and performance, such as tenderness and flavor. Abdominal fat (AF) in chickens is one of the main waste products at slaughter. Excessive AF reduces feed efficiency and carcass quality.

**Results:**

To analyze the differential deposition of IMF and AF in chickens, gene expression profiles in the breast muscle (BM) and AF tissues of 18 animals were analyzed by differential expression analysis and weighted co-expression network analysis. The results showed that IMF deposition in BM was associated with pyruvate and citric acid metabolism through *GAPDH*, *LDHA*, *GPX1*, *GBE1*, and other genes. In contrast, AF deposition was related to acetyl CoA and glycerol metabolism through *FABP1*, *ELOVL6*, *SCD*, *ADIPOQ*, and other genes. Carbohydrate metabolism plays an essential role in IMF deposition, and fatty acid and glycerol metabolism regulate AF deposition.

**Conclusion:**

This study elucidated the molecular mechanism governing IMF and AF deposition through crucial genes and signaling pathways and provided a theoretical basis for producing high-quality broilers.

**Supplementary Information:**

The online version contains supplementary material available at 10.1186/s12864-022-08538-0.

## Introduction

Fat deposition in broilers is affected by genetic factors (breed, sex, and genotype) and nongenetic factors (nutrition, age, and environmental factors). The rate of fat deposition varies across tissues in broilers. In addition, glucose and lipid metabolism and hormone sensitivity vary in adipocytes from different tissues [1–4]. The deposition of abdominal fat (AF) and intramuscular fat (IMF) is regulated by different mechanisms in chickens [5], and this characteristic allows the genetic selection of broilers with high IMF and low AF [6].

Previous studies showed a significant and positive correlation between IMF and AF [7, 8]. In contrast, a study found that the chicken with lower AF had higher IMF. The discrepancy may be caused by breeding characteristics, environmental factors, genetic selection, and sampling methods [6]. A balanced selection population (increasing IMF, decreasing AF percentage (AFP)) was more effective than selecting for IMF alone in Jing-xing yellow chicken [9]. Various genetic mechanisms regulate lipid deposition in tissues. Genes encoding perilipin and long-chain acyl CoA dehydrogenase are highly expressed in subcutaneous adipocytes, whereas genes encoding bone morphogenetic proteins 4 and 7 are abundantly expressed in intramuscular adipocytes [10]. With the development of cost-effective sequencing technologies, a large amount of transcriptome data has been generated under different biological back scenes. Currently, based on these high-throughput sequencing data, Weighted correlation network analysis (WGCNA) has been successfully applied to identify gene expression networks and biomarkers of interest in various biological domains [11–16]. Studies have shown that the transcription factor *CREB3L1* was associated with low AF, whereas the transcription factor *L3MBTL1* and the cofactor *TNIP1* were related to high IMF and low AF in the Jing-xing Huang chicken line [17]. These results underscore the need to study the genetic mechanisms regulating fat deposition in broiler chickens. Wenchang breed is mainly distributed in Hainan, the lowest altitude in south China. It is an excellent local breed with more than 400 years of breeding history. Wenchang chicken is usually marketed in 100–110 days and weighs about 1.4–1.8 kg. Wenchang chicken has the characteristics of thin and tender skin, fragrant meat, and high IMF. However, the AF content of Wenchang chicken is also high, which will cause waste in production and reduce the uniformity of population. Therefore, it is necessary to understand the characteristics of fat deposition in Wenchang chickens, which is beneficial to improve Wenchang chickens’ quality and production efficiency. Few studies jointly evaluated IMF and AF in broilers. This study evaluates differential fat deposition in breast muscle (BM) and AF tissues of Wenchang chicken, gene expression profiles these tissues based on transcriptome data, and genes and signaling pathways involved in IMF and AF deposition in chicken to reveal the difference of molecular regulation mechanisms.

## Results

### Evaluation of sequencing data

**Fig. 1:**
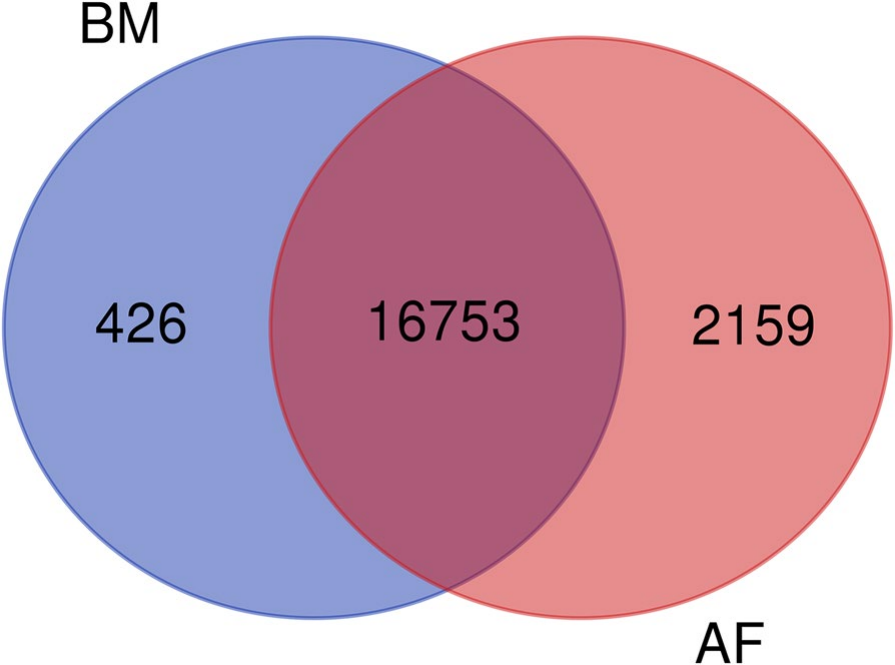
Venn diagrams of genes differentially expressed in the breast muscle and abdominal fat of broiler chickens

Transcriptomic data were obtained from the BM and AF of 18 Wenchang chickens. Thirty-six RNA-sequencing (RNA-seq) libraries were constructed and sequenced (Additional file: Table S1). A total of 23,910 genes were detected, of which 17,179 genes were expressed in BM, and 18,912 genes were expressed in AF. In addition, 72.24% (16,753/23,910) of the genes were expressed in both BM and AF (Fig. 1). Raw sequence reads from each library are shown in Additional files: Tables S2 and S3. The most genes abundantly expressed in BM and AF were glyceraldehyde-3-phosphate dehydrogenase (*GAPDH*) and fatty acid-binding protein (*FABP4*), respectively.

**Fig. 2:**
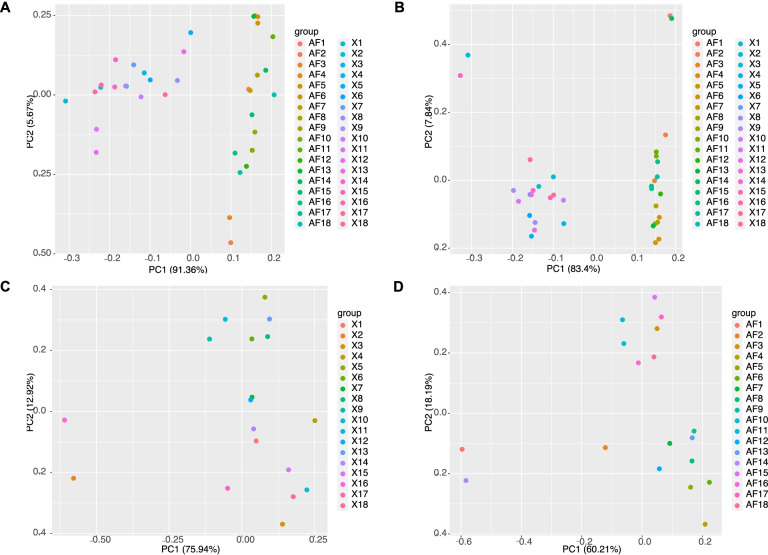
Principal component analysis (PCA) results of BM and AF tissue. **A.** PCA of phenotypes in BM and AF. **B.** PCA of genes expressed in both BM and AF. **C.** PCA of genes expressed in BM. **D.** PCA of genes expressed in AF

To explore differences in IMF and AF deposition (Table 1), Principal component analysis (PCA) of fatty acid composition was performed to assess differences in fat deposition in BM and AF tissue (Fig. 2A) [18].

**Table 1:** Phenotypes of Wenchang chicken in different tissues

Phenotypes	Breast Muscle	Abdominal Fat
IMF/AFP	1.2944 ± 0.1089^a^	4.7266 ± 0.3988^b^
TG	4.9691 ± 0.5710^a^	23.5572 ± 1.8791^b^
C10:0	ND	0.0093 ± 0.0005
C12:0	0.0364 ± 0.0023	0.0280 ± 0.0009
C14:0	0.4470 ± 0.0145	0.6762 ± 0.0117
C14:1	0.0788 ± 0.0052	0.1531 ± 0.0075
C15:0	0.0706 ± 0.0016^a^	0.08967 ± 0.0033
C16:0	26.1824 ± 0.1856^a^	27.2354 ± 0.3255^b^
C16:1	2.8540 ± 0.1824	5.1318 ± 0.2777
C17:0	0.1328 ± 0.0062	0.1417 ± 0.0060
C18:0	11.5981 ± 0.2874	6.9239 ± 0.2392
C18:1n9c	28.9134 ± 0.7301^a^	39.0340 ± 0.3500^b^
C18:2n6c	17.6387 ± 0.1924^a^	18.7408 ± 0.4955^b^
C18:3n3	0.4663 ± 0.0158	0.8446 ± 0.0158
C20:0	0.1898 ± 0.0083	0.1227 ± 0.0056
C20:1	0.2493 ± 0.0079	0.3452 ± 0.0129
C20:2	ND	0.0132 ± 0.0007
C20:3n3	0.1106 ± 0.0080^a^	0.0082 ± 0.0007^b^
C20:3n6	0.9901 ± 0.0583^a^	0.0846 ± 0.0042^b^
C20:4n6	7.8473 ± 0.4910^a^	0.1625 ± 0.0135^b^
C20:5n3	0.1186 ± 0.0073^a^	0.0082 ± 0.0009^b^
C21:0	0.4080 ± 0.0460^a^	0.1433 ± 0.0074^b^
C22:0	0.1949 ± 0.0152^a^	0.0254 ± 0.0015^b^
C22:1n9	0.1233 ± 0.0098^a^	0.0214 ± 0.0008^b^
C22:2	ND	0.0039 ± 0.0009
C22:6n3	0.8751 ± 0.0648^a^	0.0139 ± 0.0016^b^
C23:0	0.0872 ± 0.0053^a^	0.0233 ± 0.0009^b^
C24:0	0.1540 ± 0.0084^a^	0.0131 ± 0.0013^b^
C24:1	0.2333 ± 0.0130^a^	0.0027 ± 0.0008^b^

Note: Means differences of fatty acids among treatments were determined with Duncan’s multiple range test. a, b Means in the same rows with different superscripts differ (*P* < 0.05) (*n* = 18). ND means not detected

PCA analysis was performed on 16,753 genes co-expressed in BM and AF (Fig. 2B). PCA analysis was performed on all expressed genes in BM and AF, respectively (Fig. 2C and 2D).

### WGCNA identified module genes significantly related to phenotypes

**Fig. 3:**
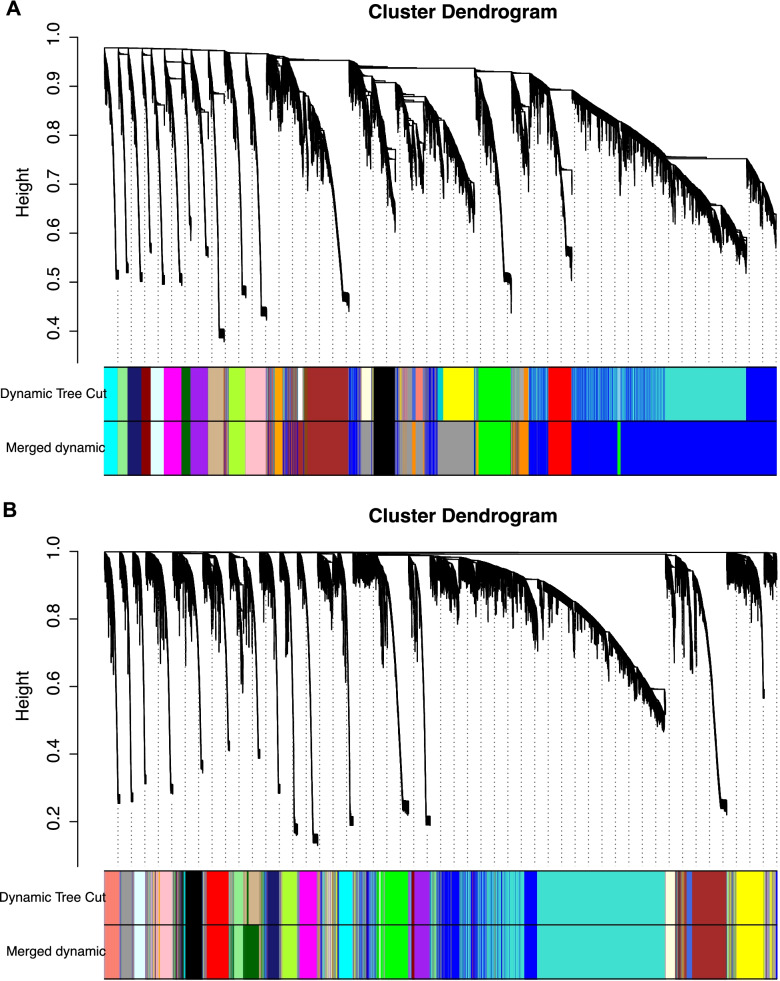
Clustering of gene modules. **A.** Breast muscle tissue microarray. **B.** Abdominal fat tissue microarray. Upper panel: genes were clustered into different groups. Lower panel: genes were assigned to modules after dynamic tree-cutting and merging

Genes with fragments per kilobase of transcript per million fragments mapped values of less than one were excluded. Gene co-expression networks were constructed using 18,295 expressed genes from BM and 19,778 expressed genes from AF, and the coordination matrix was converted to a topology matrix. The similarity in gene co-expression between modules was quantified using a dynamic tree-cutting algorithm, and modules with height values of less than 0.25 were merged (the minimum number of genes in the module was set to 30) (Fig. 3).

**Fig. 4:**
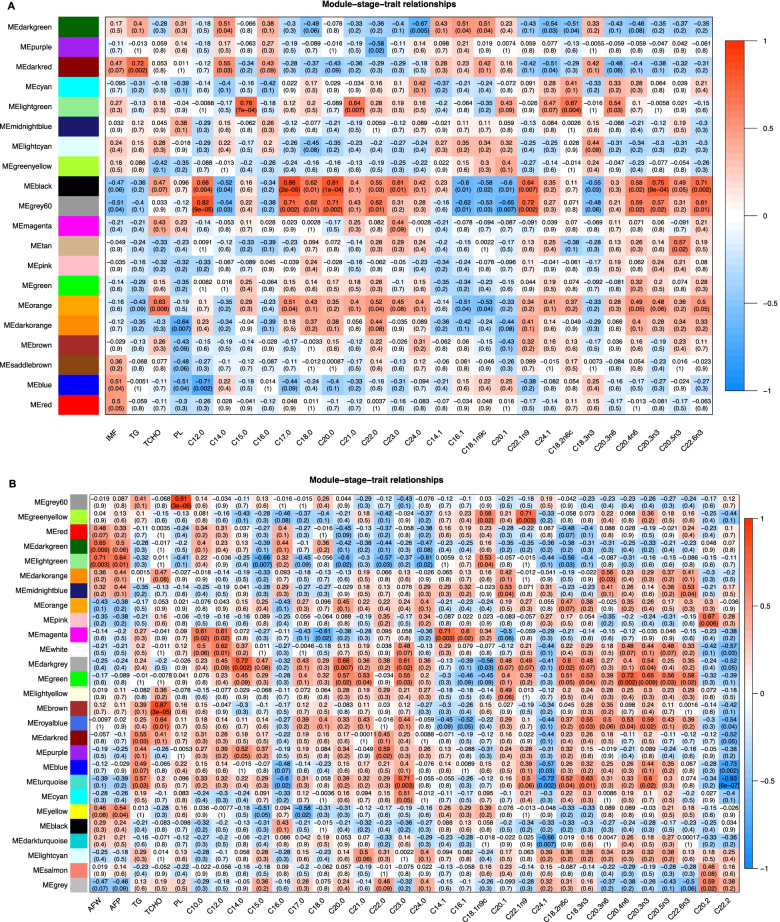
Relationship between gene modules and traits in breast muscle tissue (**A**) and abdominal fat tissue (**B**). Note: The upper value in each module is the correlation coefficient between the module and the lower character, and the lower value is the p-value of the coefficient

Twenty and twenty-seven expression modules were detected in BM (Additional file: Table S4) and AF (Additional file: Table S5), respectively (Fig. 4), and the number of genes in each network module was counted. The largest module in BM (blue) and AF (turquoise) contained 6972 and 5755 genes, respectively, and the smallest module in BM (dark brown) and AF (white) contained 113 and 47 genes, respectively.

Gene modules significantly associated with genetic traits were identified. Absolute values of 0 and 1 indicated weak and strong correlation between the module and the trait, respectively. Among the evaluated traits, IMF was significantly and positively correlated with the blue module (r = 0.51, *P* = 0.04), triglycerides (TG) was significantly and positively associated with the darkred module (r = 0.72, *P* = 0.002), total cholesterol (TCHO) was significantly and positively related to the darkorange module (r = 0.63, *P* = 0.008), phospholipids (PLIP) was significantly and positively associated with the orange and blue modules (r = -0.64, *P* = 0.007; r = -0.51, *P* = 0.04) and traits such as fatty acids composition showed significant correlations with related modules. AF weight showed a significant and positive correlation with the darkgreen and lightgreen modules (r = 0.65, *P* = 0.009; r = 0.71, *P* = 0.003), and AFP showed a significant and positive association with the lightgreen and yellow modules (r = 0.64, *P* = 0.01; r = 0.54, *P* = 0.04). In AF tissue, TG was significantly and positively correlated with the darkred and turquoise modules (r = 0.55, *P* = 0.03; r = 0.57, *P* = 0.03). TCHO showed a significant and positive association with the brown and blue modules (r = 0.87, *P* = 3e-05; r = 0.64, *P* = 0.01). PLIP was significantly and positively related to the grey60 module (r = 0.91, *P* = 3e-06), and traits such as fatty acids composition showed a significant and positive correlations with related modules.

### Identification of DEGs in BM and AF

We selected three individuals with high IMF content and three individuals with low IMF content for BM tissue's difference analysis. And we selected three individuals with high AFP and three individuals with low AFP for AF tissue's difference analysis (Additional file: Table S6). Analysis using Deseq2 detected 461 DEGs (163 upregulated and 298 downregulated) in the high and low IMF groups (Additional file: Table S7). Pathway enrichment analysis revealed that these genes were enriched in 29 pathways, mainly including metabolic pathways, MAPK signaling pathways, arachidonic acid metabolism, glycolysis/gluconeogenesis, starch, and sucrose metabolism, fructose and mannose metabolism, and other signaling pathways (Additional file: Table S8). GO analysis showed that DEGs in this group were enriched in 27 biological processes, 16 cellular components, and 12 molecular functions (Additional file: Table S9). A total of 2010 genes (1301 upregulated and 700-downregulated) were detected in the high and low AF groups (Additional file: Table S10). These genes were enriched in 59 KEGG pathways, mainly including metabolic pathways, PPAR signaling pathway, MAPK signaling pathway, tyrosine metabolism, glycerolipid metabolism, phenylalanine metabolism, glycerophospholipid metabolism, pyruvate metabolism, unsaturated fatty acid biosynthesis, fatty acid metabolism, and other signaling pathways (Additional file: Table S11). GO analysis showed that DEGs in this group were enriched in 66 biological processes, 21 cellular components, and 18 molecular functions (Additional file: Table S12).

### Identification of pathways in BM and AF

The genes expressed in the modules significantly associated with all phenotypes were selected from BM and AF tissues, respectively. The significantly expressed genes in the modules significantly associated with the traits were pooled, and a total of 3818 expressed genes were identified in BM tissue, while a total of 5826 expressed genes were identified in AF tissue.

**Fig. 5:**
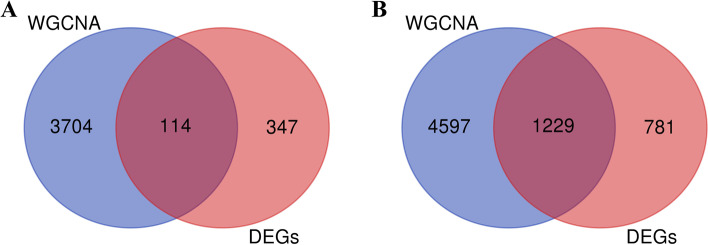
Venn of module characteristic genes and differential expression genes. **A**. Breast muscle tissue. **B**. Abdominal fat tissue

These genes were analyzed separately in association with differentially expressed genes, in which 114 co-expressed genes were obtained in BM tissue (Fig. 5A), while a total of 1229 co-expressed genes were obtained in AF tissue (Fig. 5B).

**Fig. 6:**
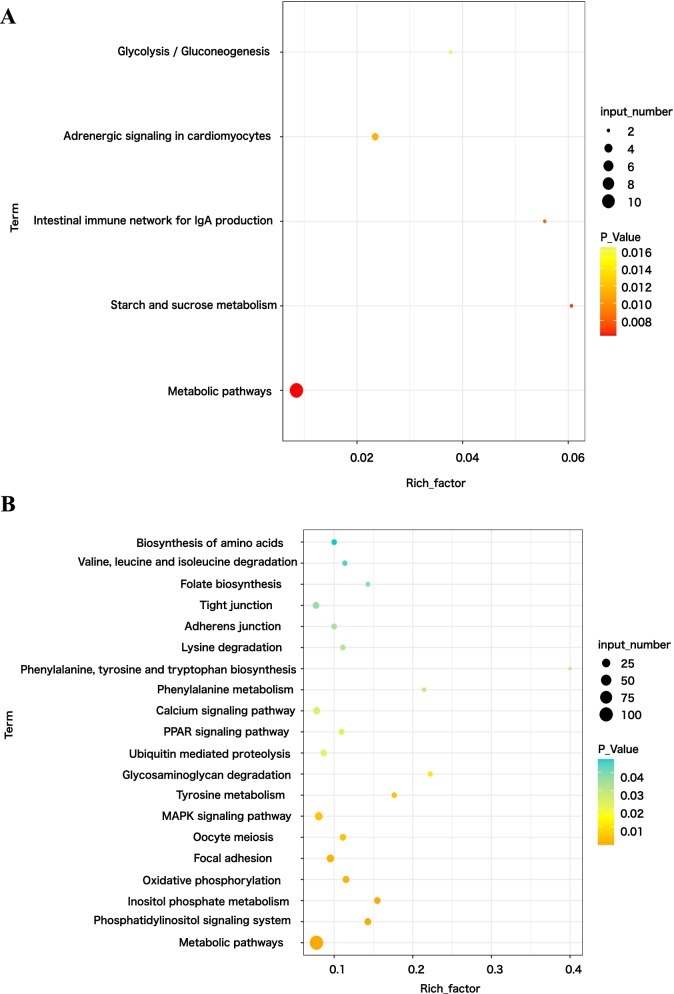
Pathway enrichment analysis of co-expression genes in breast muscle (**A**) and abdominal fat (**B**)

The differential module genes obtained from the two tissues were analyzed separately for KEGG pathway enrichment. In BM tissue, 114 genes were assigned to 5 KEGG pathways (*P* < 0.05) (Fig. 6A), and genes were enriched in MAPK signaling pathway, ECM-receptor interaction, Gap junction, Tight junction, Vascular smooth muscle contraction, GnRH signaling pathway, and Focal adhesion. In AF tissue, 1229 genes were assigned to 41 KEGG pathways (*P* < 0.05) (Fig. 6B), and genes were mainly enriched in biological processes, including Oxidative phosphorylation, ABC transporters, C-type lectin receptor signaling pathway, Phosphatidylinositol signaling system.

### Identification of co-expressed genes

**Fig. 7:**
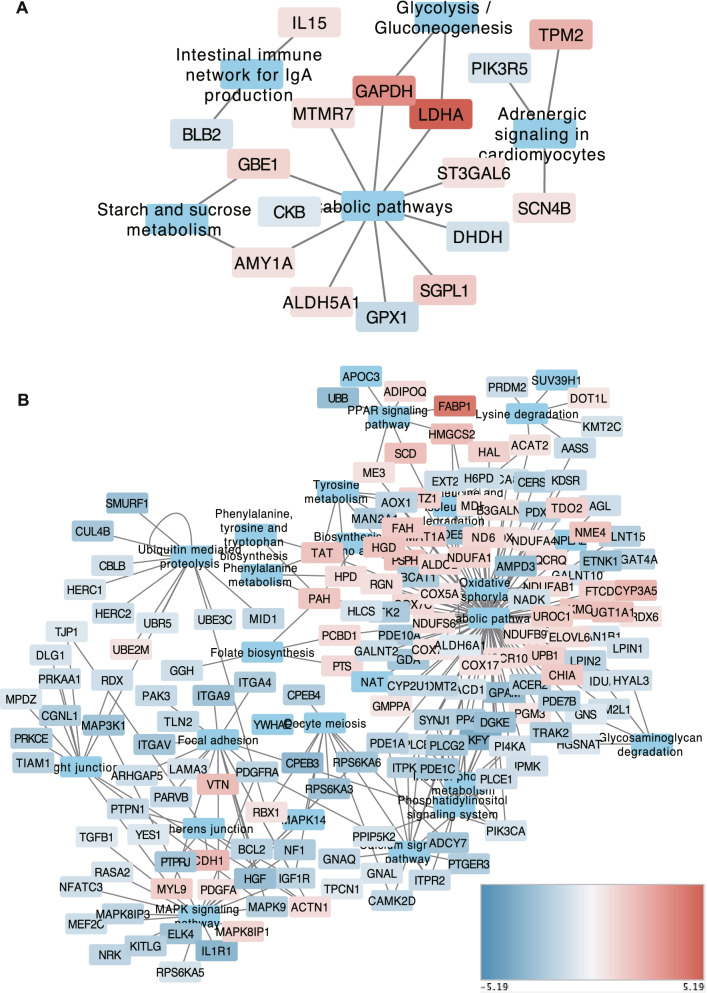
Network of pathways and genes in breast muscle tissue (**A**) and abdominal fat tissue (**B**)

The molecular expression networks between pathway and co-expressed genes in BM (Fig. 7A) and AF (Fig. 7B) were performed, respectively. The genes more abundantly expressed in BM with high IMF were related to muscle development (*TPM2*, *MTMR7*) and glycolipid metabolism (*LDHA*, *AMY1A*, *ST3GAL6*, *GBE1*, *SGPL1*, *GPX1*, *COX5A*, *ALDH5A1*). The genes more significantly expressed in the high AF group were related to fatty acid metabolism (*ELOVL6*, *SCD*, *FABP1*, *ME3*, *ADIPOQ*, *HMGCS2*, *PDGFA*, *ACAT2*).

## Discussion

Yellow feather broiler chickens are widely consumed in China, and chicken meat production has increased in recent years. IMF and AF contents strongly affect the quality of chicken meat. Fasting before slaughter is essential in the experimental sampling process [19]. The fasting method can reduce the volume of stomach contents and the risk of visceral microbial contamination during slaughter [20]. In general, moderate fasting (no more than 24 h) is suitable for meat quality because it accelerates glycogen consumption [21, 22]. Especially in poultry, fasting can decrease breast muscle glycogen and increase initial pH [23–25]. Studies have shown that fasting for a certain period before slaughter can control the muscle glycogen content of beef cattle without affecting the marbling score [26–28]. Studies have shown that fasting for 12 h has less influence on the structural changes and plasma corticosterone level and less on the energy metabolism level of chest muscle tissue [29]. Therefore, fasting for 12 h before slaughter would not significantly impact subsequent meat quality and energy metabolism levels, and the effect of glucose metabolism in food on the chicken could be ruled out.

Although the mechanisms underlying fat deposition in broiler chickens are well known, differences in the rate of fat deposition in BM and AF tissues have not been determined.

PCA results based on phenotypes showed a strong separation of breast muscle tissue from abdominal fat tissue, as expected. This suggests that BM tissue and AF tissue have different regulatory roles in phenotype. Many DEGs can be identified by RNA-seq analysis, however, the characterization of gene expression patterns and correlation with phenotypes are challenging, underscoring the need to perform weighted co-expression network analyses. In the study, DEGs were identified in BM and AF by RNA-seq analysis and joint analysis with WGCNA to identify specifically expressed genes and signaling pathways for IMF and AF deposition. The molecular regulatory mechanisms of fat deposition in different tissues at the same age were elucidated in chicken.

Based on the differentially expressed genes, the results of KEGG signaling pathway analysis showed that DEGs from BM and AF tissues were jointly enriched in the AGE-RAGE signaling pathway, amino acid biosynthesis, cytokine-cytokine receptor interactions, local adhesion, GnRH signaling pathway, MAPK signaling pathway, metabolic pathway, in diabetic complications. Tight junctions and other signaling pathways. Studies have shown that MAPK signaling pathway is involved in lipid deposition [30, 31], which can regulate PPAR pathway [32]. Moreover, PPAR signaling pathway also has the function of regulating lipid metabolism [33, 34].

In contrast, DEGs in BM tissue were specifically enriched in pathways related to gluconeogenesis (glycolysis/gluconeogenesis, starch, sucrose metabolism, fructose, mannose metabolism, pentose, and glucuronide). *LDHA* and *LDHB* in the glycolysis/gluconeogenesis signaling pathway are involved in pyruvate metabolism and tricarboxylic acid cycle, facilitating the glycolytic process by converting pyruvate to lactate [35]. In addition, *GBE1* is essential in starch and glycogen formation metabolism, and there is a strong link between the hexokinase family genes *HK1*, *HK2*, *HK3* in this pathway and the metabolic process of glucose. These data suggest that IMF deposition in BM tissue mainly depends on glucose metabolism, with some energy metabolism involved in the process.

DEGs in BM tissue were specifically enriched in pathways related to fatty acid syntheses (PPAR signaling, glycolipid metabolism, glycerophospholipid metabolism, and unsaturated fatty acid biosynthesis). Sixteen genes were enriched in the PPAR signaling pathway, including *HMGCS2* (catalyze ketogenesis) [36, 37], *ACOX1* (lipid degradation) [38], *ADIPOQ* (adipocyte differentiation) [39], *APOA1* and *ME3* (cholesterol metabolism) [40], *SCD* (fatty acid transporter proteins) [41], *PLIN1* and *PLIN2* (lipid droplet protection) [42–44], *ACSL1* [45], *FABP7*, *FABP1*, *FADS2*. The formation of intracellular lipid droplets is a highly conserved process, including fatty acid transport and activation, neutral lipid synthesis, and lipid droplet formation, regulated by many factors and pathways [46]. These results suggest that AF deposition is dependent on fatty acid synthesis and transport and lipid droplet formation.

WGCNA was performed using RNA-seq data to identify expressed genes in different modules and predict the role of genes in lipid deposition. Several modules were significantly associated with IMF/AFW/AFP, lipid composition, and fatty acids metabolism in BM and AF, and the expressed genes significantly associated with the phenotypes in the modules were aggregated together and analyzed jointly with DEGs, which could further identify the functional genes that play essential roles in the IMF and AF deposition. Among all expressed genes, *GAPDH* has the highest expression level in breast muscle tissue. *GAPDH* is a key enzyme in glycolysis. It plays an important role in glycolysis by catalyzing the first step of this pathway by converting D-glyceraldehyde 3-phosphate (G3P) to 3-phospho-d-glyceroylphosphate [47, 48]. *FABP4* gene in the lipid-binding protein family regulates fatty acid uptake and transport [41].

We identified 114 expressed genes associated with Metabolic pathways, Starch and sucrose metabolism, Intestinal immune network for IgA production, Adrenergic signaling in cardiomyocytes, and Glycolysis/Gluconeogenesis in BM. These pathways are regulated by genes such as *ALDH5A1*, *LDHA*, *GAPDH*, *GBE1*, and *GPX1* and may play roles in IMF deposition. A total of 1229 expressed genes associated with 41 signaling pathways were identified in AF, of which 20 pathways that may play roles in AF deposition via PPAR signaling pathway, amino acid biosynthesis, oxidative phosphorylation, may be controlled by several genes, including *ADIPOQ*, *ELOVL6*, *HMGCS2*, *ME3*, *DGKE*, *AOX1*, *UBB*, *SCD*, *APOC3*, and *FABP1*. The results revealed that IMF deposition in BM tissue was regulated by gluconeogenesis-related pathways (glycolysis/gluconeogenesis, starch and sucrose metabolism signaling pathways), and by several genes, including *GAPDH*, *LDHA*, *GPX1*, *ALDH5A1*, and *GBE1*, among which, the key differential gene *ALDH5A1* can catalyze a step in the degradation of the inhibitory neurotransmitter γ-aminobutyric acid [49]. The protein encoded by *LDHA* is involved in pyruvate metabolism by catalyzing the conversion of lactate to pyruvate in anaerobic glycolysis. *GAPDH* plays an important role in glycerol metabolism. These data further suggest that IMF deposition in BM is dependent on gluconeogenesis and energy metabolism.

While expressed genes in AF tissue are enriched to PPAR signaling pathway, oxidative phosphorylation, amino acid biosynthesis, and other signaling pathways, acting through genes such as *FABP1*, *ELOVL6*, *SCD*, and *ADIPOQ*. A PPAR signaling pathway is involved in lipid droplets formation and mitochondrial metabolism, and fatty acid oxidation and lipid synthesis are necessary for cellular signaling [50]. *FABP1* plays a crucial role in the PPAR pathway, *PPARG* expression, and fatty acid uptake, transport, and metabolism in vivo [51]. In addition, *PPARϒ* can be regulated by modulating *SCD1* expression to control fatty acid synthesis in adipocytes [52]. *ELOVLs*, which encode long-chain and extra-long-chain fatty acid elongases, play an important role in synthesizing fatty acids and can limit elongation. *ELOVL6* is involved in synthesizing fatty acid enzymes in vivo, promoting fatty acid elongation [53]. *ACAT2* encodes acetyl-coenzyme A acetyltransferase 2, which is involved in acetyl-coenzyme A metabolism [54]. These results suggest that AF deposition may result from changes in fatty acid synthesis through mitochondrial activity.

The results suggest that different signaling pathways regulate fat deposition in BM and AF tissues. IMF deposition in BM was associated with pyruvate and citrate metabolism through *GAPDH*, *LDHA*, *GPX1*, *GBE1*, and other genes, whereas AF deposition was related to acetyl-coenzyme A and glycerophospholipid metabolism through *FABP1*, *ELOVL6*, *SCD*, and *ADIPOQ*. The transcriptional regulation of genes in network modules associated with traits and metabolic pathway analysis can provide new insights into the genetic mechanisms governing fat deposition in broiler chickens.

## Conclusion

The analysis of transcriptome data from BM and AF tissues, combined with differential expression analysis and WGCNA, showed that the genes *GAPDH*, *LDHA*, *GPX1*, and *GBE1* were involved in regulating IMF deposition, and the genes *FABP1*, *ELOVL6*, *SCD*, and *ADIPOQ* determined AF deposition. Glycolysis/gluconeogenesis and other signaling pathways play an important role in IMF deposition, whereas PPAR metabolism controls AF deposition. Therefore, we hypothesized that IMF deposition in BM tissue may affect energy metabolism during myocyte gluconeogenesis while fatty acid synthesis pathways may affect AF deposition.

## Materials and methods

### Animals and Sample Collection

Wenchang chickens (18 females) of 98 days were obtained from the Institute of Poultry Research of the Chinese Academy of Agricultural Sciences (Yangzhou, China). The diet (Additional file: Table S13) was formulated according to the feeding standard (NY/T33-2004). Under the same standard conditions of light (20 lx), temperature (35℃ ~ 37℃), humidity (not less than 50%), and immunization schedule, after a 12-h fast, the chickens were stunned by electric shock and killed by cervical dislocation at 98 days. After slaughter, breast muscle (BM) and AF were collected and immediately stored at − 80 °C until use.

### Measurement of Biochemical Indices [18]

The determination of fatty acids in BM and AF tissues refers to the national standard《GB/T 5413.27–2010 Food Safety National Standard for infant food and dairy products determination of fatty acids》in the first method: acetyl chloride—methanol methyl esterification method. The determination of intramuscular fat in BM tissue refers to the national standard《GB/T 5009.6–2016 National Standard for Food Safety determination of fat in food》in the first method: Soxhlet extraction. The TG, TCHO, and PLIP contents in BM and AF tissues were measured using assay kits (Nanjing Jiancheng Bioengineering Institute, Nanjing, China, and Beijing Leichuang Biotechnology Co., LTD, China). We weighed 2.0 ± 0.01 g breast muscle and abdominal fat samples and mixed them with 18 mL anhydrous ethanol in 50 ml centrifuge tubes. Each sample was homogenized by a hand-held high-speed homogenizer (35,000 r/min, 15 s/ time). The samples were crushed, centrifuged at 2500 r/min for 10 min. After centrifugation, the supernatant was used for measurement. Refer to the instruction for specific sample loading quantity. After incubation, the absorbance value (510 nm) was measured using a microplate reader (Tecan Infinite 200 Pro, Switzerland).

### Transcriptome Profiling

Transcriptome profiling of 18 breast muscle and 18 abdominal fat tissues was performed on the Illumina PE150 platform (Berry Genomics; Beijing, China) (https://www.berrygenomics.com). RNA was extracted as described previously [55]. Adapter sequences and other low-quality data were removed using Cutadapt. Reads were aligned to the reference genome using HISAT, while clean reads obtained by filtering were compared to the reference genome according to the gene position information specified in the genome annotation file gtf, and the total comparison rate was approximately 90%.

### Differential Expression Analysis

Based on phenotypic data, DEGs were identified in BM tissue with high IMF and low IMF (from three animals each) and AF tissue with high AF and low AF (from three animals each) using DESeq2 in R [56]. Adjusted p-values (q-values) were calculated using Benjamin and Hochberg’s approach for controlling the false discovery rate. Genes with | log2(fold change) |≥ 0.58 and *P* < 0.05 were considered differentially expressed.

### Weighted Gene Co-expression Network Analysis (WGCNA)

WGCNA was performed using the WGCNA package [57] with default settings and minor modifications. For the analysis of genes expressed in BM tissue (*n* = 16), the minModuleSize was set to 50, and mergeCutHeight was set to 0.25 for tissue- or stage-specific module detection (soft threshold = 4). For the analysis of genes expressed in AF tissue (*n* = 15), the minModuleSize was set to 30, and mergeCutHeight was set to 0.25 for tissue- or stage-specific module detection (soft threshold = 10). Dendrograms were created using topology overlap matrix, module detection, and similar module merging functions. Module-trait relationships were calculated, and correlations were adjusted using Benjamin-Hochberg correction.

### Pathway Enrichment Analyses

Gene ontology (GO) enrichment analysis was performed with the DAVID database (https://david.ncifcrf.gov/) to identify gene classes and categories. Kyoto Encyclopedia of Genes and Genomes (KEGG) pathway enrichment analysis was performed using KOBAS version 3.0 (http://kobas.cbi.pku.edu.cn). The significance level was set at *P* < 0.05. The gene-pathway interaction networks for the candidate genes were visualized with Cytoscape 3.8.0 (http://www.cytoscape.org/) [58].

### Statistical Analyses

The significance of differences between means was assessed using Student’s t-test in SPSS 22.0 (IBM Corp, Armonk, NY, USA). *P*-values less than 0.05 were considered statistically significant. Data are presented as the mean ± standard error of the mean.

## Supplementary Information

**Additional file 1.**

## Data Availability

The RNA sequencing raw data reported in this paper have been deposited in the Genome Sequence Archive [59] in BIG Data Center [60] under accession number CRA006031, which can be publicly accessed at http://bigd.big.ac.cn/gsa. All data analyzed during this study are included in the article and additional files.

## Abbreviations

ACAT2: Acetyl-CoA Acetyltransferase 2
ACOX1: Acyl-CoA Oxidase 1
ACSL1: Acyl-CoA Synthetase Long Chain Family Member 1
ADIPOQ: Adiponectin, C1Q And Collagen Domain Containing; AF: Abdominal fat; AFW: Abdominal fat weight
AFP: Abdominal fat percentage
ALDH5A1: Aldehyde Dehydrogenase 5 Family Member A1
AMY1A: Amylase Alpha 1A
AOX1: Aldehyde Oxidase 1
APOA1: Apolipoprotein A1
APOC3: Apolipoprotein C3
BM: Breast Muscle
COX5A: Cytochrome C Oxidase Subunit 5A
CREB3L1: CAMP Responsive Element Binding Protein 3 Like 1
DEGs: Differential Expressed Genes
DGKE: Diacylglycerol Kinase Epsilon
ECM: Extracellular Matrix
ELOVL6: ELOVL Fatty Acid Elongase 6
FABP1: Fatty Acid Binding Protein 1
FABP4: Fatty Acid Binding Protein 4
FABP7: Fatty Acid Binding Protein 7
FADS2: Fatty Acid Desaturase 2
GAPDH: Glyceraldehyde-3-Phosphate Dehydrogenase
GBE1: 1,4-Alpha-Glucan Branching Enzyme 1
GO: Gene Ontology
GPX1: Glutathione Peroxidase 1
HK1: Hexokinase 1
HK2: Hexokinase 2
HK3: Hexokinase 3
HMGCS2: 3-Hydroxy-3-Methylglutaryl-CoA Synthase 2
IMF: Intramuscular Fat
KEGG: Kyoto Encyclopedia of Genes and Genomes
L3MBTL1: Histone Methyl-Lysine Binding Protein 1
LDHA: Lactate Dehydrogenase A
ME3: Malic Enzyme 3
PCA: Principal Component Analysis
PDGFA: Platelet Derived Growth Factor Subunit A
PLIN1: Perilipin 1
PLIN2: Perilipin 2
PLIP: Phospholipid
RNA-Seq: RNA-sequencing
SCD: Stearoyl-CoA Desaturase
SGPL1: Sphingosine-1-Phosphate Lyase 1
ST3GAL6: ST3 Beta-Galactoside Alpha-2,3-Sialyltransferase 6
TCHO: Total Cholesterol
TG: Triglycerides
TNP1: Transition Protein 1
UBB: Ubiquitin B
WGCNA: Weighted Gene Co-expression Network Analysis
